# Efficacy of the newly discovered entomopathogenic nematode *Steinernema adamsi* against *Helicoverpa zea*: life stage susceptibility, UV tolerance, and field performance

**DOI:** 10.2478/jofnem-2025-0012

**Published:** 2025-04-24

**Authors:** James Paul Glover, Nathan Spaulding, Justin George, Maribel Portilla, Gadi V.P. Reddy, Adler Dillman

**Affiliations:** USDA-ARS, Southern Insect Management Research Unit, Stoneville, MS 38776, USA; Department of Nematology, UC Riverside, CA 92521, USA

**Keywords:** *Helicoverpa zea*, IPM, entomopathogenic nematode, biological control, field efficacy

## Abstract

*Helicoverpa zea* is a major agricultural pest, particularly in cotton, and poses significant challenges due to its ability to develop resistance to chemical insecticides. This study evaluates the efficacy of the entomopathogenic nematode (*Steinernema adamsi*) and its mutualistic bacteria (*Xenorhabdus*) as biological control agents against *H. zea* larvae in both laboratory and field settings. In laboratory assays, mortality rates for 1^st^ to 4^th^ instars were high, ranging from 74.2% to 100%, while 5^th^ instars exhibited significantly lower susceptibility (<37% mortality). Pupae were completely resistant to nematode infection. The impact of UV radiation on nematode efficacy was assessed, with mortality decreasing from 100% in control conditions (0 hours of UV exposure) to 71.8% after 5 hours of UV exposure, highlighting the vulnerability of *S. adamsi* to UV degradation. In addition, *Xenorhabdus* caused 100% mortality in *H. zea* larvae when injected directly into the hemocoel, but oral toxicity was significantly lower, with 36% mortality in 7 days post-exposure. Field experiments demonstrated that the combination of *S. adamsi* with 0.05% sodium alginate (hygroscopic agent) and 0.02% Congo red (UV protectant) resulted in a significant increase in larval mortality. In field test A, where *S. adamsi* was applied in water, mortality averaged 56% with 82% EPN infection. In field test B, the combined treatment of sodium alginate and Congo red led to 98% larval mortality, although infection rates were lower and statistically non-significant. The addition of these protective agents likely enhanced the environmental stability and efficacy of the nematodes under field conditions. These findings suggest that *S. adamsi* can be an effective biological control agent for *H. zea*, particularly when combined with formulations that protect against UV radiation and desiccation. Future research should focus on optimizing nematode delivery systems to improve field efficacy under diverse environmental conditions.

## 1. Introduction

The corn earworm (*Helicoverpa zea*) is a significant pest affecting cotton (*Gossypium hirsutum* L.) and other row crops, including corn (*Zea mays* L.), and soybean (*Glycine max* [L.] Merrill) crops worldwide, including the Mississippi Delta (Luttrell and Jackson, 2012). This pest is well reported for its wide host range, high reproductive capacity, and ability to develop resistance to chemical insecticides, posing significant challenges to agricultural production and pest management (Fitt, 1989; Jackson et al., 2008). In the Mississippi Delta, where cotton remains a crucial economic crop, the corn earworm has been a persistent economic pest, causing extensive damage to cotton and corn fields and reducing yields (Arends, 2022). Given its economic importance and the growing concerns over pesticide resistance, there is an urgent need for alternative, sustainable pest control strategies (Yang, 2019, 2020; Tabashnik and Carriere, 2017; Reisig and Kurtz, 2018).

Entomopathogenic nematodes (EPNs) have emerged as a promising biological control agent against a variety of insect pests, including the corn earworm (Cabanillas et al., 1996, 1995; Bong, 1983, 1986). EPNs, primarily from the genera *Steinernema* and *Heterorhabditis*, are natural enemies of many insect species and are characterized by their ability to infect and kill hosts with symbiotic bacteria (Shapiro-Ilan et al., 2017). These nematodes search for insect hosts in the soil, penetrate them through natural openings, and release bacteria that quickly multiply and kill the insect, allowing the nematodes to reproduce (Kaya et al., 2006, Shapiro-Ilan et al., 2009; Kaya and Stock, 1997). Due to their broad host range and ability to infect most insects inhabiting similar environments, with few exceptions demonstrating host specificity nematodes have become attractive alternatives to chemical pesticides, which can have negative effects on the environment and human health (Kaya and Gaugler, 1993). EPNs are considered an environmentally friendly option for integrated pest management (Lacey et al., 2015, 2012). In addition, they do not pose a risk of developing resistance in pest populations (Stock and Goodrich-Blair, 2012).

The application of entomopathogenic nematodes in agricultural settings has gained attraction due to their versatility and effectiveness in controlling a wide range of pests, including white grubs, root weevils, and cutworms (Kaya and Gaugler, 1993; Shapiro-Ilan et al., 2010). They have also been used successfully against soil-dwelling pests such as corn rootworms and wireworms (Stock and Goodrich-Blair, 2012; Sandhi et al., 2020). Entomopathogenic nematodes have been shown to reduce insect pest populations and increase crop yields in various agricultural settings (Kaya and Gaugler, 1993; Feaster and Steinkraus, 1996). Studies have shown that EPNs can be effectively applied in field crops, orchards, and greenhouse settings to manage pests such as root weevils, cutworms, and thrips (Mahmoud, 2017). In cotton fields, EPNs have demonstrated potential as a biological control strategy to reduce earworm populations, especially when integrated with other pest management practices (Dillman et al., 2012). The development of formulations and delivery systems that enhance the survival and infectivity of EPNs in field conditions, such as encapsulation techniques and the use of UV protectants, has further improved their efficacy and adoption in pest control programs (Grewal et al., 2012; Shapiro-Ilan et. al., 2015).

However, the effectiveness of EPNs for aboveground applications has been limited due to desiccation (Navaneethan et al., 2010) and ultraviolet radiation (Gaugler et al., 1992; Gaugler and Boush, 1978). To combat these environmental stressors, spray adjuvants, stickers, and promoters have been tested in combination to enhance the performance of EPNs (Glazer, 1992; Glazer et al., 1992; Macvean et al., 1982; Noodidum et al., 2016). This study aims to evaluate the efficacy of the newly discovered entomopathogenic nematode species, *Steinernema adamsi,* isolated initially from the soil of a logan tree (*Dimocarpus sp*.) in the Mueang Lamphun District of Thailand using traditional baiting techniques (Baniya et al. 2024). Here, we test formulations for controlling corn earworm populations in the Mississippi Delta.

The objectives of this study were to 1) evaluate the virulence and efficacy of the newly discovered entomopathogenic nematode *S. adamsi* against *Helicoverpa zea,* 2) evaluate the effect of ultraviolet degradation on *S. adamsi* against *H. zea*, (3) test the efficacy of *S. adamsi* mutualistic bacteria *Xenorhabdus* through hemocoel injections and oral toxicity, and (4) test the efficacy of *S. adamsi* against *Helicoverpa zea* in the field under ecologically relevant environmental conditions in blooming cotton.

## 2. Material and Methods

### 2.1. Insect rearing

*Helicoverpa zea* colony used in experiments was originally field collected in late spring 2023 from several counties in the Mississippi Delta from Crimson clover, *Trifolium incarnatum* L., using a 38.1 cm (15″) diameter sweep net. The research colony was maintained on an artificial soybean and wheat germ-based diet developed for *Heliothis virescens* for four successive generations at USDA-ARS, Insect Management Research Unit, Stoneville, MS, USA (Blanco et al., 2009). Insect colonies were reared at 27°C in environmental chambers with a photoperiod of 14:10 h (L:D) and 75% RH. Larval instars used for experiments were 1–2 d old, with an assumed equal sex ratio.

### 2.2. Entomopathogenic nematode rearing

The *S. adamsi* EPN strain was originally obtained from the Department of Nematology, University of California, Riverside. The EPNs were reared in the last instars of the greater wax moth, *Galleria mellonella* L. (Lepidoptera: Pyralidae) (Josh’s Frogs, Owosso, Michigan). The waxworms were reared according to Zhang et al. (2024) briefly. Insects were inoculated at a ratio of ten infective juveniles (IJs) per host in a Petri dish lined with a single layer of VWR filter paper. One mL of an EPN suspension, containing 100 IJs/mL, was added to each dish, along with ten hosts, then covered with black plastic bags and incubated at room temperature (~22°C). After approximately 48 hours, once the hosts had succumbed, the cadavers were transferred to White traps (Kaya and Stock, 1997), kept at room temperature, and covered with a black plastic bag. The traps were checked daily for IJ emergence, and emerging IJs were collected and stored in tissue culture flasks at 13°C. Prior to each experiment, IJs were counted under a dissecting microscope using counting slides (Chalex, LLC, Park City, Utah, USA), and suspensions with the desired EPN concentration were prepared accordingly.

### 2.3. Laboratory experiments

#### 2.3.1. Efficacy of *S. adamsi* on 1^st^–5^th^ instars and pupae of *H. zea*

The EPN strain *S. adamsi* was tested against first through fifth instars of 3-day-old *H. zea* larvae and pupae. The EPN strain was replicated four times for each developmental stage with 30 insects per replication. A single insect was placed in a diet cup containing diet (see above), and a one ml suspension of EPNs at the rate of 100 IJ/ml was pipetted into the dorsal surface of the larvae, while control larvae received 1 ml of water. The diet cups were then placed into an environmental chamber and incubated at 27°C photoperiod of 00:24 h (L:D) and 75% RH. Experimental insects with positive indications of EPN infection were recorded daily until control insects developed to the next instar. Percentage mortality is referred to as the number of dead larvae divided by the total number of larvae used reported as a percentage.

#### 2.3.2. Effect of ultraviolet radiation on *S. adamsi* against *H. zea*

The *S. adamsi* entomopathogenic nematode (EPN) strain was exposed to ultraviolet (UV) light for 0 (control covered with foil), 3 h, 5 h, and 8 h, then applied to second instar *H. zea*. A high-performance benchtop UV transilluminator (UVP Model M-15V Benchtop UV Transilluminator, 302 nm, Analytik Jena, Upland, CA) was used to expose EPN’s to ultraviolet radiation of approximately 5000 UV-A/B and 60 UV-C for the prescribed treatment time. After exposure, its efficacy was tested on 3-day-old second-instar *H. zea* larvae. The experiment was conducted with four replicates, each containing 30 insects, and was repeated twice. Each larva was placed individually in a diet cup with prepared diet. A one-milliliter suspension of UV-exposed EPNs, at a concentration of 100 infective juveniles (IJ) per milliliter, was applied over and around the insect, making contact with the dorsal side of the larvae and surrounding diet. In contrast, control larvae received 1 ml of water. The diet cups were then placed in an environmental chamber set to 27°C, with a photoperiod of 00:24 h (L:D) and 75% relative humidity. The larvae were monitored daily for signs of EPN infection until the control group molted to the next instar. Mortality was determined by dividing the number of dead larvae by the total number of larvae used.

#### 2.3.3. Efficacy of *S. adamsi* mutualistic bacteria *Xenorhabdus* using hemocoel injections and oral toxicity

The EPN *S. adamsi’s* mutualistic bacteria *Xenorhabdus* was isolated from hemolymph bleed wax worms with positive EPN infections and cultured on triphenyl tetrazolium chloride supplemented tergitol-7-agar (Dunphy & Webster, 1985; Shapiro-Ilan and Gaugler, 2002). Bacteria were subcultured at 14 d intervals and maintained at 27°C on tergitol-7-agar. Bacterial suspensions used for bioassays were grown in submerged Luria-Bertani broth for 3 days. Fourth instar *H. zea* were used to test the efficacy of *Xenorhabdus* mutualistic bacteria via hemocoel injections using 10μl injections and oral toxicity. Individuals were cradled between the thumb and forefinger, where a 10μl syringe (Hamilton Microliter syringe, 701 ASN, volume 10 μL, needle size 23s ga) was inserted between the A5 and A6 abdominal segments, and a 10μl of bacterial suspension was injected. Insects were maintained in individual diet-rearing cups for the duration of the assay. Oral toxicity bioassays were conducted using the same bacterial suspensions (see above), where individual caterpillars contained within a single diet cup had the complete surface topically coated with 200μl. The diet cups were then placed in an environmental chamber set to 27°C in darkness with 75% relative humidity. The larvae were monitored daily for characteristic signs of infection (yellow to brown coloration) until the control group molted to the next instar. Mortality was determined by dividing the number of dead larvae by the total number of larvae used.

### 2.4. Field experiments

#### 2.4.1. Experimental design for field experiments with *S. adamsi* against *H. zea*

Field experiments with the EPN strain *S. adamsi* were tested against 3-day-old first instar *H. zea* larvae in cotton blooms at the USDA-ARS research farm located in Leland, Mississippi. Experiments were conducted on blooming cotton planted in strips of non-Bt (Deltapine 1822XF, Bayer CropScience, St. Louis, MO). The hygroscopic agent and a UV protectant assayed in this series of field experiments was 0.05% sodium alginate (Sigma Aldrich, W201502, St. Louis, MO) and 0.02% Congo red (Sigma Aldrich, C6277, St. Louis, MO) in addition to a water carrier. Sodium alginate or algin is a naturally occurring, food-safe polysaccharide found in brown algae. Algin is extremely hydrophilic and forms a viscous gum when hydrated. Its salts are known as alginates in the presence of metals such as sodium and calcium. Congo red is a well-established UV protectant with many uses in histology and microscopy for staining cell walls of plants and fungi and Gram-negative bacteria (Shapiro and Robertson, 1990; Acar and Sipes, 2022). Field temperature and UV index were measured and recorded at the time of application from approximately 7:00 AM to 8:00 AM, using a digital thermometer (AcuRite Brand) and digital UV A/B meter to measure solar radiation. EPN treatments and controls (sprayed with water) were replicated five times; each replication consisted of twenty randomly selected first position-one-day-old white flowers.

**Figure 1:**
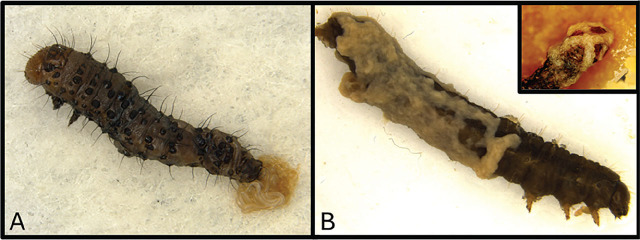
Stages of *S. adamsi* Infection in *H. zea* Larvae. (A) Second instar *Helicoverpa zea* larva showing a positive infection of *Steinernema adamsi* through the anus. (B) Fourth instar *H. zea* larva with a large EPN mat covering most of its body, indicating advanced nematode infection. The upper right inset image shows a *H. zea* larva with a severe infection of the head capsule by *S. adamsi*.

**Figure 2:**
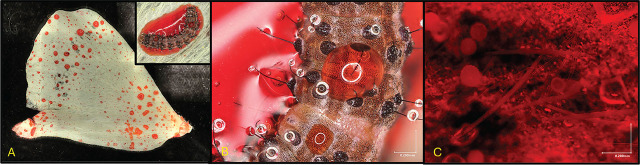
Application of Alginate/Congo Red Encapsulated *S. adamsi* Nematodes on *H. zea* larvae in Cotton Blooms. (A) Three-day-old *Helicoverpa zea* larva inside a cotton bloom, demonstrating infestation conditions. (B) Detailed view of an alginate/Congo red treatment droplet applied to the dorsal surface of the larva. (C) Magnified image showing encapsulated *Steinernema adamsi* nematodes within the alginate/Congo red droplet.

One-day-old white blooms were individually infested with 3-day-old first instar corn earworm larvae in the early morning using a fine-tip paintbrush and immediately sprayed with formulations. Flowers were physically separated >2m from adjoining treatment combinations to prevent any cross-contamination. Sixty ml pump sprayers (Crafters Square, city, state) were used for the application of 1 ml aliquots of *S. adamsi* formulations (Zhang et al., 2024). Cotton flowers treated with EPNs were collected 24 h after artificial infestation and individually searched using a jeweler’s headlamp magnification tool to locate larvae. Experimental larvae were then placed individually in diet cups with 0.5 ml of germ-based diet (see above), incubated at 27°C in environmental chambers with a photoperiod of 14:10 h (L:D) and 75% RH. Insects were observed daily for mortality up to 72 h. Dead larvae were collected and dissected under a compound light microscope to determine EPN infection status (Figs. 1A,B). Experimental field testing of the EPN strain *S. adamsi* for its sensitivity to desiccation from high temperatures and UV radiation experiments were conducted on days with full sun and little to no cloud cover in experimental plots. Field test A (initial efficacy test) commenced August 29, 2024, when cotton plants were in the fourth week of bloom. The EPN treatments consisted of (i) *S. adamsi* suspended in water and applied at a rate of 10 IJ’s per cm^2^. They were used to assay 3-day-old first-instar *H. zea* larvae. Field test B (encapsulation and UV degradation test) was run on September 4, 2024, when cotton plants were in the fifth week of bloom. The EPN treatments consisted of (i) *S. adamsi* suspended in 0.5% sodium alginate and 0.02% Congo red applied at a rate of 50 IJ’s per cm^2^ and used to assay 3-day old first instar *H. zea* larvae (Fig. 2).

### 2.5 Statistical analysis

Original percentages were normalized using arcsine square root transformation to stabilize variances and approximate normality before performing the analysis of variance (ANOVA) (Richter and Fuxa, 1990). Post-hoc means were separated in SAS version 9.4 using Tukey HSD (α < 0.05). Untransformed treatment means are presented for ease of interpretation.

## 3. Results

### 3.1. Laboratory experiments

#### 3.1.1. Effect of ten EPN strains on 1^st^–5^th^ instars, and pupae of *H. zea*

In this series of laboratory bioassays, mortality from EPN infection was significantly higher at 3 d for all instars than in the control group (insects topically applied with water) across this study (Table 1). Experimental data showed that the EPN *S. adamsi* infection in eggs of *H. zea* (data not shown) was considerably low, 0.5%. Laboratory assays with more than 400 *H. zea* eggs yielded only 2 eggs with visible EPN infections and had a single EPN inside the individual egg. Significant variation was observed for instar mortality of *H. zea* at 3 d post topical application ranging from 74.2% to 100%. Mortality for 1^st^ through 3^rd^ instars was statistically similar causing on average 76.4% mortality. Conversely, mortality observed for fourth instars was 100% across the study, while larger 5^th^ instar *H. zea* larvae were the least susceptible instar (<37%) to the EPN *Steinernema adamsi*. Pupae of *H. zea* never became infected with the EPN *S. adamsi* across the entire study (Table 1).

**Table 1:** Mortality (% ± SEM) of 1^st^-5^th^ instars, and pupae of *Helicoverpa. zea* caused by *Steinernema adamsi* entomopathogenic nematode strain in the laboratory (27°C, 75% RH). Mean values within a column followed by the same letter are not significantly different at *P* < 0.05 (Tukey’s HSD test).

EP^N^ strain	*H. zea*					

	1^st^ instar	2^nd^ instar	3^rd^ instar	4^th^ instar	5^th^ instars	Pupae
*S. adamsi*	75.83 ± 4.7^a^	74.21 ± 1.6^a^	79.16 ± 2.3^a^	100^b^10 ± 1.2^d^	36.66 ± 3.6^c^	0^d^
Water	0^d^	0^d^	0^d^		0^d^	0^d^
*F*-Value_(1, 7)_	353.76	62.57	166.20	1019.97	87.96	-
*P*-Value	<.0001	<.0002	0.0001	<.0001	<.0001	-

#### 3.1.2. Effect of ultraviolet degradation on *S. adamsi* against *H. zea*

**Figure 3:**
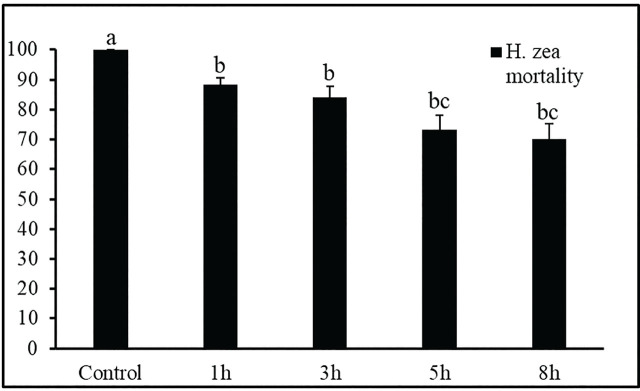
Mortality (% ± SEM) of 2^nd^ instars of *Helicoverpa. zea* caused by *Steinernema adamsi* exposed to ultraviolet radiation in the laboratory (27°C, 75% RH). Means with different letters are significantly different via Tukey’s HSD test after a significant ANOVA, *p* = 0.05).

The impact of UV radiation from a benchtop transilluminator on the *S. adamsi* strain was assessed by exposing the nematode strain to UV for 0, 3, 5, and 8 h, followed by testing against second instar *H. zea* larvae (Fig. 3). In the control group (0 hours UV exposure), 100% mortality was observed. After 1 hour and 3 hours of UV exposure, mortality rates were similar with approximately 86.2% mortality. Mortality rates were statistically similar for the 5 h and 8 h UV degradation times, with a reduction to 71.8%. The efficacy of EPNs after exposure to UV radiation after 3 hours was reduced by 13.8% when compared to controls, and a 16.7% reduction in efficacy was observed after 5 h (*F* = 1102.44; df = 3, 7; *P* = < 0.0001).

#### 3.1.3. Efficacy of *S. adamsi* mutualistic bacteria *Xenorhabdus* using hemocoel injections and oral toxicity

The efficacy of the mutualistic bacteria *Xenorhabdus* spp. associated with *S. adamsi* was evaluated through hemocoel injections and oral toxicity assays against *H. zea* larvae. In the hemocoel injection bioassay, 100% mortality was observed within 48 hours post-injection, demonstrating the high virulence of the bacterial symbiont when directly introduced into the insect hemolymph (Table 2). In contrast, the oral toxicity bioassays where *Xenorhabdus* bacteria was administered to the larvae via diet exhibited a lower but statistically significant mortality rate of 36% 7 d post-exposure. The average mortality ranged from 3.4% to 46.7%, for the bacterial concentration tested. While oral exposure resulted in delayed mortality compared to hemocoel injections, significant insecticidal effects were still observed, suggesting that *Xenorhabdus* bacteria remains toxic when ingested and has reduced efficacy compared to direct injection.

**Table 2:** Mortality (% ± SEM) of 2^nd^ instars of *Helicoverpa. zea* caused by *Steinernema adamsi* mutualistic bacteria *Xenorhabdus* hemocoel injections and oral toxicity in the laboratory (27°C, 75% RH).

Exposure method	% Mortality ± SEM
*Hemocoel injections* Water	5^th^ instar
	100^a^
	0^b^
	F_(1, 4)_ = 69.45
	*P* < 0.0001
*Oral toxicity* Water	2^nd^ instar
	35.93 ± 1.5^a^
	2.83 ± 0.87^b^
	F_(1, 4)_ = 35.04
	*P* < 0.0274

Mean values within a column followed by the same letter are not significantly different at *P* > 0.05 (Tukey’s HSD test).

### 3.2. Field experiments

#### 3.2.1. *S. adamsi* EPN strain in combination with a hygroscopic agent and a UV protectant against *H. zea* larvae in cotton blooms

**Figure 4:**
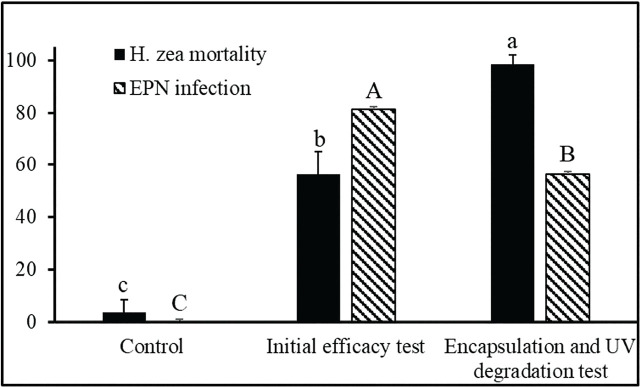
Effect of *Steinernema adamsi* formulations from field experiments A and B on 1^st^ instars of *Helicoverpa zea* in cotton (2024, Leland, Mississippi). Field experiment A (Initial efficacy test) was conducted on August 29, 2024, with an average 33°C, 62%RH and under full sun (9495/29) (U.V. AB/C) using water as the carrier agent at 10 IJs/cm^2^. Field experiment B (Encapsulation and UV degradation test) was conducted on September 4, 2024, with an average 34°C, 55%RH, and full sun (9289/33) (U.V. AB/C) using alginate (0.05%, w/v) and Congo red (0.02%, w/v) formulations at 50 IJs/cm^2^. Means with different letters are significantly different via Tukey’s HSD test after a significant ANOVA, *p* = 0.05).

Field experiments were conducted to evaluate the efficacy of *S. adamsi* combined with a hygroscopic agent (0.05% sodium alginate) and a UV protectant (0.02% Congo red) against 3-day-old 1^th^ *H. zea* larvae in cotton blooms. Two separate field tests were performed under full sun conditions at the USDA-ARS research farm in Leland, Mississippi. In field test A (initial efficacy test) conducted on August 29, 2024, *S. adamsi* was applied in water at the rate of 10 infective juveniles (IJs) per cm^2^. This treatment resulted in an average mortality of 56% in *H. zea* larvae compared to controls (*F* = 60.17; df = 1, 6; *P* = < 0.0015) (Fig. 4). The percentage of larvae infected by *S. adamsi*, confirmed through dissection, was approximately 82%, indicating effective penetration and infection under these conditions (*F* = 440.30; df = 1, 6; *P* = < 0.0001). In field test B (encapsulation and UV degradation test) conducted on September 4, 2024, a significantly higher concentration of *S. adamsi* (50 IJs per cm^2^) was applied in combination with 0.05% sodium alginate and 0.02% Congo red histological dye. This treatment combination significantly increased mortality with 98% larval mortality (*F* = 91.66; df = 1, 6; *P* = < 0.0002) (Fig. 4). However, EPN infection rates were markedly lower and not statistically significant compared to field test A (initial efficacy test). The combination of sodium alginate and Congo red formulation increased overall mortality, it may not have enhanced nematode infection rates statistically (*P* > 0.05). Overall, the combined use of sodium alginate and Congo red in field test B (encapsulation and UV degradation test) significantly enhanced the mortality rates of *H. zea* larvae, likely due to the improved environmental stability of the nematodes. However, the decrease in EPN infection percentages compared to the water-based application in field test A (initial efficacy test) indicates that the combination treatment’s effectiveness may be largely attributed to increased desiccation and UV protection rather than enhanced nematode infectivity.

## 4. Discussion

This study highlights the efficacy of the newly discovered entomopathogenic nematode *S. adamsi* (EPN) strain in controlling *H. zea* larvae under both laboratory and field conditions while also exploring the effects of UV radiation and the role of mutualistic bacteria in causing insect mortality. Laboratory experiments demonstrated that EPN infection significantly increased larval mortality across all instars, excluding eggs and pupae, compared to controls, with mortality rates ranging from 74.2% to 100% for 1^st^ to 4^th^ instars. However, the 5^th^ instar larvae were more resistant to infection, exhibiting only 37% mortality, a result consistent with previous studies that observed decreased susceptibility in later larval stages due to increased body size and developed immune systems (Acharya et al. 2020). Corn earworm pupae were completely resistant to *S. adamsi* infection, which is likely attributed to their well-developed cuticle and reduced metabolic activity during this life stage (Kaya and Gaugler, 1993).

The UV radiation experiments demonstrated that the UV exposure significantly impacted the efficacy of *S. adamsi*. In the control group (0 hours UV exposure), 100% mortality was observed in *H. zea* larvae. However, after 3 h of UV exposure, a 13.8% reduction in efficacy was observed, with further reductions of 16.7% after 5 h of exposure. Shapiro-Ilan et al. (2006), reported similar decreases in nematode viability and infectivity after prolonged exposure to UV radiation. The UV degradation likely damaged the nematode cuticle, reducing its ability to successfully penetrate and infect the larvae (Smits, 1996). Similarly, other *Steinernema* spp. have shown increased resilience to UV exposure and desiccation. Ramakrishnan et al. (2022) explored the effects of rapid desiccation on EPNs and found that infective juveniles of *Steinernema carpocapsae* were more adaptable and tolerant, requiring RH of > 74%, compared to an optimal RH of > 90% for *Steinernema feltiae* or *Heterorhabditis bacteriophora* species.

The mutualistic bacteria *Xenorhabdus* spp. associated with *S. adamsi* also exhibited potential insecticidal effects, particularly when injected directly into the hemocoel of *H. zea* larvae. In the hemocoel injection bioassays, 100% mortality was observed within 48 hours, consistent with the rapid virulence of *Xenorhabdus* when introduced into the insect hemolymph (Forst and Nealson, 1996). However, oral administration of *Xenorhabdus* resulted in significantly lower mortality rates (36% at 7 days post-exposure), indicating that while the bacteria retain toxicity when ingested, their efficacy is diminished compared to direct injection. These results are comparable with previous studies, which have shown that the gut environment can degrade bacterial toxins, reducing their effectiveness when consumed orally (Dowling et al., 2007).

Shapiro-llan and Goolsby (2021) and Mazurkiewicz et al. (2021) conducted field studies evaluating various UV protectants and encapsulation agents and found Barricade® fire gel and AtpolanBio 80 EC, provided a moisture barrier that enhanced the persistence of nematodes on plant surfaces. Field experiments provided further insights into the practical application of *S. adamsi* for pest control in an agricultural setting. In field test A (initial efficacy test), where *S. adamsi* was applied in water at a rate of 10 IJs per cm^2^, a 56% mortality rate was observed, with 82% of larvae showing EPN infection. This suggests that even at lower concentrations, the nematodes were effective in infecting and killing *H. zea* larvae. However, in field test B (encapsulation and UV degradation test), where a higher concentration of nematodes (50 IJs per cm^2^) was combined with sodium alginate and Congo red, mortality rates increased to 98%, though the percentage of infected larvae decreased. The significant improvement in mortality, despite the lower infection rates, suggests that the combination of alginate and Congo red may have enhanced the environmental stability of the nematodes, as both agents are known to protect biological control agents from desiccation and UV degradation (Walia et al. 2008).

Overall, these findings highlight the potential of *S. adamsi* as an effective biological control agent against *H. zea* larvae, particularly when combined with protective agents such as sodium alginate and Congo red. The enhanced efficacy of the combined treatment in the field demonstrates the importance of optimizing environmental conditions to ensure nematode survival and performance. Future research should focus on further refining nematode formulations and delivery systems to maximize their effectiveness in diverse agricultural environments.
